# Liquid Crystal Elastomers Filaments for Adaptive Textiles and Soft Robotics: A Processing‐Centric Review

**DOI:** 10.1002/marc.70307

**Published:** 2026-05-23

**Authors:** Anne Schwarz‐Pfeiffer, Shazia Mehtab

**Affiliations:** ^1^ Faculty of Textile and Clothing Technology Hochschule Niederrhein University of Applied Sciences Department Mönchengladbach Germany

**Keywords:** extrusion, filament fabrication, liquid crystal elastomers (LCEs), process standardization, RM257, RM82, soft actuators, UV crosslinking

## Abstract

Liquid crystal elastomers (LCEs) combine mesogenic orientational order with rubber elasticity, enabling large, reversible actuation for applications in soft robotics, smart textiles, artificial muscles, and adaptive structures. However, translating LCEs from lab‐scale films to continuous, industrially relevant filaments remains constrained by fragmented and non‐standardized processing strategies. This review presents an integrated synthesis‐to‐filament framework linking molecular formulation, rheological conditioning, flow‐induced alignment, and network fixation. Benchmark systems based on diacrylate mesogens (RM82/257) and triazine crosslinkers (TATATO) are highlighted, where staged Michael‐addition produces shear‐thinning oligomers (30–200 Pa s) suitable for extrusion, wet spinning, and melt drawing. Distinct viscosity regimes (low, intermediate, and high) are systematically compared, clarifying their effects on filament stability, alignment retention, and actuation performance. We further examine how mesogen architecture, crosslinker geometry, and photopolymerization kinetics define processable viscosity windows and defect‐free filament formation. The coupled interplay between viscosity, extrusion pressure, and flow rate is emphasized to establish realistic and scalable processing windows. Processing strategies, including nozzle design, draw ratios, in‐line UV curing, and thermal annealing, are outlined for encoding anisotropy and improving durability. The review concludes with a roadmap for standardized processing metrics, sustainable chemistries, and emerging machine learning‐assisted formulation strategies to enable scalable LCE filament manufacturing.

## Graphical abstract

Liquid crystal elastomer (LCE) filaments are fabricated through extrusion with in‐process UV curing, enabling controlled flow alignment and network locking of mesogens. The resulting structure responds to thermal or optical stimuli, inducing molecular reorientation and macroscopic contraction. This approach enables scalable production of programmable, stimuli‐responsive LCE filaments for soft actuators and adaptive structures.

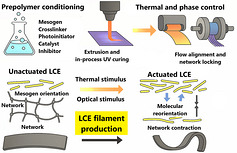

## 1 Introduction to Liquid Crystal Elastomers

Liquid crystal elastomers (LCEs) uniquely integrate three central concepts: orientational order within soft amorphous materials, stimulus‐responsive molecular configurations [1], and permanently fixed topological constraints [2]. Understanding the chemical and physical behavior of macromolecules is fundamental for designing responsive polymer systems. A key challenge is designing responsive materials that can imitate the functions of biological systems. Achieving this requires materials that are both highly mobile and capable of maintaining their shape with qualities that traditional polymers in their glassy or semi‐crystalline forms cannot provide [3]. Over the past three decades, LCEs have evolved from fundamental physical curiosities into one of the most promising classes of soft, multifunctional materials for actuation, sensing, and programmable mechanics [1, 2, 4]. Their unique ability to couple mesogenic orientational order with polymer chain anisotropy enables reversible, large‐amplitude shape changes under external stimuli such as heat, light, or electric fields [5, 6]. This coupling translates microscopic rearrangements of mesogens into macroscopic deformations, producing reversible bending, twisting, contraction, and even three‐dimensional shape morphing [7, 8, 9].

Such versatility makes LCEs attractive for emerging technologies, including soft robotics [10], artificial muscles [11], adaptive optics [12], haptic interfaces, and wearable systems that mimic biological motion [13, 14]. Despite significant progress in molecular design and actuation performance, the transition from laboratory‐scale synthesis to industrially viable filament production remains limited [15, 16, 17]. This gap arises primarily from the absence of standardized processing methodologies and comparable rheological metrics across studies. In this context, the concept of a processing window is defined here as the range of rheological, thermal, and kinetic conditions under which LCE precursors can be extruded. This review addresses these challenges by consolidating formulation, processing, and structure relationships by establishing a unified framework for filament fabrication. In addition, emerging data‐driven approaches, including machine learning‐assisted formulation optimization, are highlighted as promising tools to overcome multiparametric design barriers in LCES systems.

## 2 Prepolymer Formulation Design

### 2.1 Resin Synthesis

**FIGURE 1:**
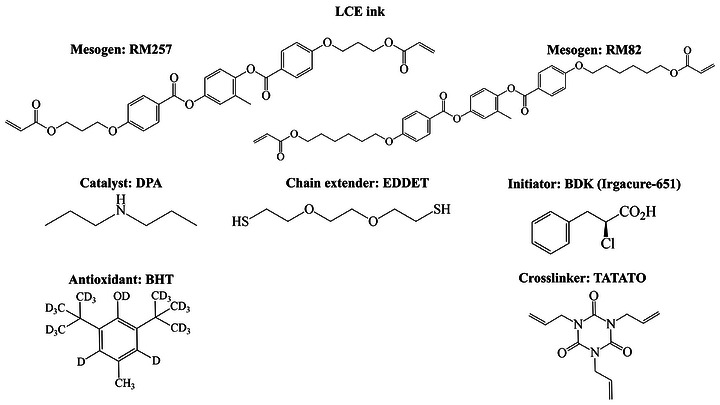
Chemical structures of the components to formulate the LCES ink, including the reactive mesogens RM82 and RM257, the crosslinker TATATO, the Michael‐addition catalyst DPA, the chain extender EDDET, the photoinitiator Irgacure‐651 (BDK), and the antioxidant BHT [22, 23].

Designing LCE formulations for filament fabrication requires the co‐optimization of mesogenic order, melt rheology, and curing kinetics. As filament manufacturing demands controlled flow, alignment retention, and robust post‐curing, the formulation must sustain liquid‐crystalline phase stability while maintaining viscosities appropriate for extrusion, wet spinning, or continuous UV drawing. Contemporary fiber‐grade systems achieve this through diacrylate mesogens (e.g., RM82, RM257), multifunctional allyl crosslinkers such as TATATO, thiol chain extenders (EDDET), tertiary‐amine Michael catalysts, and free‐radical photoinitiators (Table 1). This composition enables a two‐stage polymerization pathway, Michael‐addition oligomerization followed by UV crosslinking to stabilize flow‐induced alignment (5,18,6). As illustrated in Figure 1, representative embodiments include the FibeRobo continuous drawing platform, which employs RM82‐EDDET‐TATATO formulations combined with Irgacure‐651 and BHT to produce hundreds of meters of sub‐millimeter fibers with reliable reversible contraction under thermal loading [18, 19].

**TABLE 1:** Benchmark LCES filament chemistries (representative literature values).

System	Actuation strain (%)	Stress (MPa)	Cycles to 50% decay	Refs.
RM82‐TATATO	42 ± 3	0.35	>1000	[11]
RM257‐TATATO	28 ± 2	0.55	600	[14]
Main‐chain DDA‐9	110 ± 10	0.15	100	[20]
Thiol‐acrylate	35 ± 4	0.25	800	[21]

### 2.2 Mesogens

Among commercially available thermotropic reactive mesogens, the diacrylates RM82 and RM257 remain the most widely used benchmarks in the formation of polymer‐stabilized and crosslinked liquid‐crystal networks. RM82, comprising a flexible biphenyl core and long hexyloxy spacers, exhibits a broad nematic temperature range, relatively low glass‐transition temperature, and adequate viscosity that make it highly suitable for filament fabrication, continuous extrusion, and uniform alignment locking. In contrast, RM257 contains a rigid α‐methylstilbene mesogenic core, which imparts stronger orientational order and higher clearing temperatures, but also leads to stiffer and more brittle polymer networks under high crosslink densities. These contrasting molecular architectures have established RM82 as the preferred mesogen for processability‐driven applications, while RM257 is favored where high mechanical rigidity and strong mesogenic coupling are required [21, 24, 25] (Table 2).

**TABLE 2:** Comparative physical properties of representative reactive mesogens used in LCES filament fabrication.

Monomer	Chemical name/formula	Functional group	*T*_n__i_ (°C)	*T*_g_ (°C)	Mechanical flexibility	Typical use	Refs.
RM82	4‐*bis*[4‐(6‐acryloyloxyhexyloxy)benzoyloxy]‐2‐methylbenzene (C_39_H_44_O_10_)	Diacrylate	90–100	15–25	Moderate	Balanced performance; ideal for filament extrusion.	[25]
RM257	1,4‐*bis*[4‐(3‐acryloyloxypropyloxy)benzoyloxy]‐2‐methylbenzene (C_33_H_32_O_10_)	Diacrylate	110–125	25–35	Brittle	High order; suitable for rigid elastomers/actuators.	[26]
RM105	4‐methoxyphenyl 4‐((6‐(acryloyloxy)hexyl)oxy)benzoate (C_23_H_26_O_6_)	Monoacrylate	70–80	10–20	Flexible	Blending agent for increased compliance.	[27]
RM23	4‐cyanophenyl 4‐((6‐(acryloyloxy)hexyl)oxy)benzoate (C_22_H_21_NO_4_)	Monoacrylate	90–110	20–30	Moderate	Used in dielectric‐tunable & reprocessable LC systems.	[28]

**FIGURE 2:**
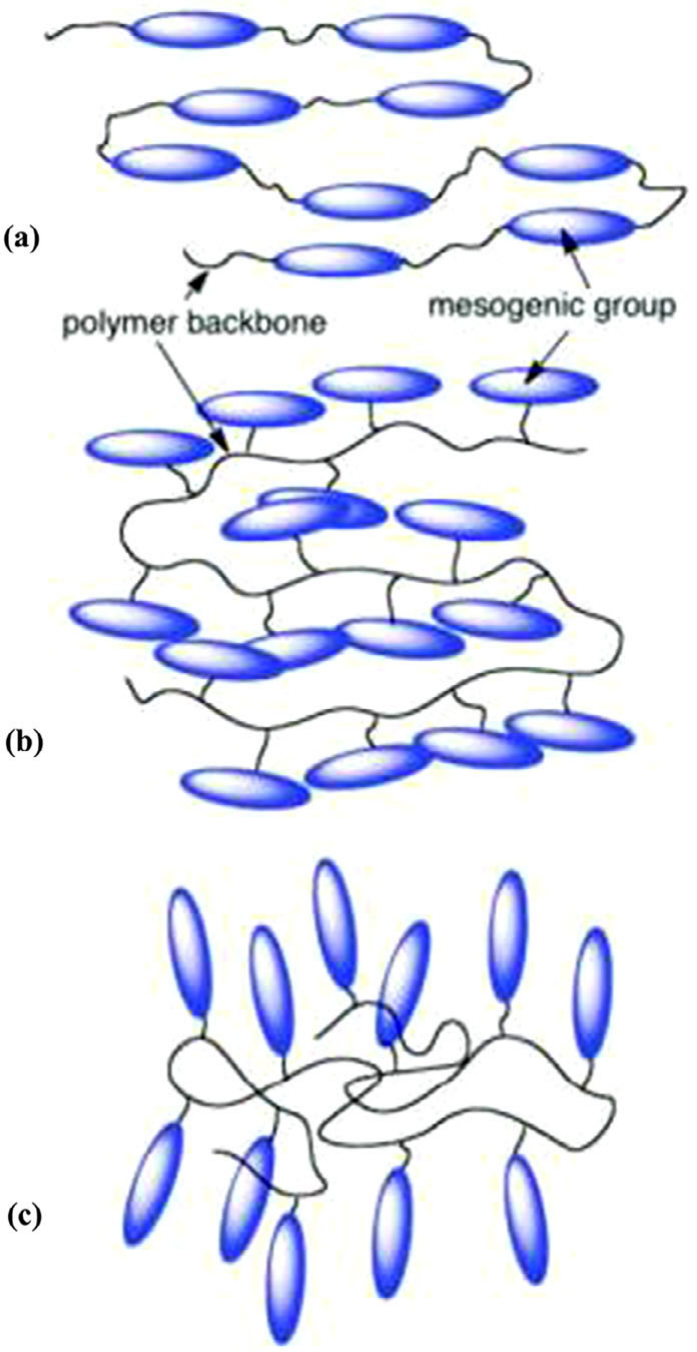
Schematic of (a) end‐on main chain, (b) side‐on side chain, and (c) end‐on side chain LCEs. Reprinted with permission from Ref. [29]. Copyright 2017, American Chemical Society. License number: 6265840807917.

Side‐chain polysiloxane LCEs, initially developed by Finkelmann and later reviewed comprehensively by Warner and Terentjevin 2003, exhibit very low glass‐transition temperatures of approximately 5°C, high backbone flexibility, and pronounced soft elasticity because the Si–O backbone efficiently transmits mesogen alignment. As illustrated schematically in Figure 2, mesogens may be incorporated into a polymer either as pendant side‐chain units or as segments within the main chain, and the end‐on or side‐on attachment geometries significantly influence backbone anisotropy and the development of nematic or smectic order (Figure 2a,b and, c) [2, 5, 24, 29]. Acrylate and methacrylate side‐chain LCEs typically display higher glass‐transition temperatures of 50°C or above and reduced soft elasticity due to the comparatively rigid carbon–carbon backbone. Additional architectural considerations arise in main‐chain LCEs, where mesogenic units are incorporated directly into the polymer backbone. End‐on main‐chain configurations 2c provide strong orientational coupling but frequently lead to elevated clearing temperatures or the formation of smectic or crystalline phases, whereas side‐on main‐chain architectures moderate this rigidity by orienting the mesogen laterally, improving conformational flexibility while preserving high orientational order [2, 5, 29].

Typical main‐chain nematic liquid‐crystal elastomers (MC‐NLCEs), such as DDA‐9 (diacrylate dimer with nine‐carbon spacers) and AZA‐9 (azobenzene diacrylate with nine‐carbon spacers), employ long ─(CH_2_)*n*‐polymethylene spacers to balance the rod‐like mesogenic order with the chain entropy required for elastomeric behavior [5, 30, 31, 32]. While such MC‐NLCES systems can deliver exceptionally large spontaneous strains, they frequently require molecular “softening” strategies‐such as long aliphatic spacers, kinked or bent‐core mesogens, or lateral substituents‐to suppress overly high nematic‐isotropic transition temperatures and to avoid unwanted smectic or crystalline phases [1, 33]. For this reason, side‐chain liquid‐crystal elastomers based on the commercial mesogens RM82 (2‐methyl‐1,4‐phenylene *bis*(4‐(6acryloyloxyhexyloxy)benzoate))and RM257 (1,4‐*bis*[4‐(3‐acryloyloxypropyloxy)benzoyloxy]benzene) remain the most scalable and reliable for filament fabrication and additive manufacturing processes [6, 34]. In contrast, more elaborate MC‐NLCES architectures are typically reserved for advanced actuator applications where their extremely large strain outputs justify the increased synthetic complexity [26, 35].

### 2.3 Crosslinkers

**FIGURE 3:**
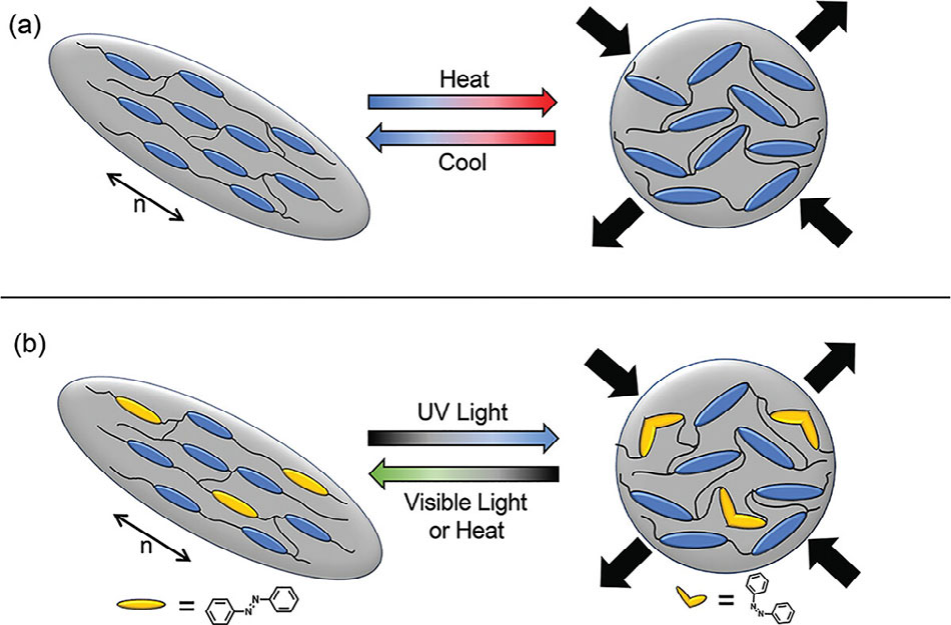
Schematic illustrations of (a) thermomechanical and (b) photomechanical deformation of liquid crystal elastomers arising from stimulus‐induced disruption of nematic order within a crosslinked polymer network. Reproduced from T. S. Hebner [38], under the terms of the Creative Commons Attribution (CC‐BY) license.

Crosslinkers dictate the network topology of LCEs, determining how mesogenic domains translate microscopic orientational order into macroscopic elastic response. Their selection governs modulus, actuation strain, fatigue resistance, creep behavior, and thermal cycling stability, making crosslink chemistry one of the most critical variables in LCES filament design [5, 6, 36, 37]. As illustrated in Figure 3a,b, respectively, thermomechanical and photomechanical actuation in LCEs arise from stimulus‐induced disruption of nematic order within a permanently crosslinked polymer network, converting microscopic orientational changes into macroscopic deformation.

From a materials perspective, crosslinkers can be broadly grouped into mesogenic diacrylates, flexible aliphatic diacrylates, multifunctional acrylates, thiol‐based click crosslinkers, and allyl triazine derivatives, each offering distinct trade‐offs between soft elasticity, nematic coupling, and curing kinetics [30, 32, 39]. Historically, the Freiburg school demonstrated that allyl‐based crosslinkers, particularly triallyl triazine‐trione (TATATO), form exceptionally homogeneous elastomer networks capable of retaining monodomain nematic order under large deformation [24, 32]. The rigid triazine heterocycle with three symmetrically positioned allyl moieties produces an isotropic crosslink geometry that allows mechanical stretching without reorientational collapse [38]. This architecture underpinned the first generation of single‐domain LCEs and remains foundational in recent filamentary systems [16, 40, 41]. Current filament manufacturing studies reaffirm this advantage. RM82‐TATATO networks display stable flow rheology, predictable gelation behavior, and robust alignment retention across extrusion, wet spinning, and UV‐drawing strategies [42, 43, 44]. In the FibeRobo continuous fiber platform, this formulation locks shear‐induced alignment while enabling reversibility across long fiber lengths, producing sub‐millimeter LCES filaments with consistent thermal actuation at textile scale [18, 19]. In woven LCES fabrics, the same network structure maintains uniform anisotropy after post‐curing, enabling programmable bending, buckling, and pattern‐selective morphing without progressive network degradation [22, 45, 46].

Alternative crosslinker chemistries offer useful mechanical tuning but impose constraints on filament actuation. Flexible aliphatic diacrylates such as PEGDA or HDDA reduce *T*_g_ and increase ductility, improving extrudability and fracture tolerance [6, 45, 47]. However, at elevated loadings, they disrupt mesogenic packing, decrease nematic coupling, and attenuate contraction amplitude, making them suitable primarily as secondary modifiers. Multifunctional acrylates such as TMPTA or PETA increase crosslink density and creep resistance but suppress soft elasticity, reducing reversible strain and long‐term fatigue performance [47, 48, 49]. Thiol‐based click networks offer controlled oligomer formation and reproducible viscosity windows. Multifunctional thiols such as PETMP enable two‐stage curing‐Michael‐addition prepolymerization followed by UV‐induced network locking‐with high shape fidelity and strong printability advantages [33, 37, 49]. However, their mechanics can deviate from classical nematic elastomer behavior if polymerization kinetics are not carefully matched to mesogen alignment [50, 51, 52]. Vitrimeric crosslinkers based on dynamic covalent exchange further introduce reprocessability and shape reconfiguration, but require careful control of exchange rates to avoid actuation drift under long‐term thermal cycling [36, 53]. Beyond permanent network architectures, the introduction of dynamic covalent exchange has enabled reprocessability and shape reconfiguration in LCE systems. A landmark development in this domain was reported by Ji et al. [54], who first introduced the vitrimer concept into LCEs. By utilizing transesterification‐based dynamic chemistry, these materials can undergo network topology rearrangement at elevated temperatures, allowing for post‐polymerization programming of complex 3D shapes and surface welding while maintaining liquid crystalline order.

The influence of crosslink geometry extends beyond static elasticity and directly governs electromechanical and photothermal coupling in liquid crystal elastomer composites. In electrothermally driven semi‐crystalline LCEs/carbon‐nanotube fibers, covalent crosslinking stabilizes the nematic alignment while providing mechanical continuity between the polymer matrix and conductive CNT networks. This arrangement facilitates efficient Joule heating, spatially uniform actuation, and suppression of creep during cyclic loading, enabling large reversible strokes and high specific work output in fiber actuators designed for wearable and robotic applications [13, 55, 56]. In CNT‐reinforced LCEs laminates and braided yarns, crosslink density also controls interfacial frictional losses and prevents micro‐debonding between CNT layers, improving strain persistence and fatigue resistance under repeated bending and twisting [22, 57].

Crosslink architecture is similarly critical for optical LCEs‐CNT and LCES conductive fiber systems. In coaxially extruded waveguide fibers, a stable crosslink network maintains core–shell dimensional integrity and suppresses scattering at the CNT‐polymer interface, enabling low optical attenuation and reversible photothermal contraction over many actuation cycles [58, 59, 60]. These findings demonstrate that crosslinkers do not simply define bulk modulus but actively mediate energy transport and conversion processes that couple molecular alignment to optical, electrical, or thermal stimuli [56, 61, 62]. Crosslinker chemistry also dictates additive compatibility and interfacial robustness. Polymerizable graphene oxide‐LCEs fibers generated by covalently modifying graphene oxide sheets with reactive vinyl or acrylate groups incorporate the filler into the network topology rather than relying on physical dispersion. Covalent incorporation mitigates filler migration and stress‐concentration sites, producing more stable interfaces, improved fatigue resistance, and high volumetric work densities in NIR‐driven actuators [63, 64, 65]. By contrast, weakly bound particulate fillers embedded in loosely crosslinked matrices often delaminate under cyclic strain or photothermal heating, leading to reduced durability and progressive loss of anisotropy‐a failure mode repeatedly reported in early physically doped LCEs composites [31, 52, 66].

Across these case studies, converging evidence indicates a consistent design principle for filament‐grade LCEs. Allyl‐based triazine crosslinkers such as TATATO most reliably preserve mesogen alignment and support scalable processing, making them ideal for continuous fiber drawing, weaving, and actuator integration [18, 32, 37, 41, 43]. Flexible diacrylates and thiol‐based systems remain valuable for viscosity control and toughness enhancement but must be incorporated judiciously to avoid undermining nematic coupling. As manufacturing advances toward industrial throughput, RM82‐TATATO frameworks, optionally modified with limited compliant co‐crosslinkers or vitrimeric motifs, provide the most reproducible path toward durable, high‐performance filaments suitable for textile, robotic, and multimodal actuator deployment [5, 6, 9, 17].

### 2.4 Photoinitiator/Catalyst

Photoinitiators and catalysts regulate the chemical pathways that convert liquid crystal elastomer precursors into crosslinked networks. In photopolymerizable LCES systems, photoinitiators absorb ultraviolet or visible light and convert this energy into reactive radical or cationic species that initiate polymerization of acrylate, methacrylate, or thiol‐ene functionalities [6, 67]. The photoinitiator absorption band and quantum efficiency directly affect curing depth and the spatial distribution of crosslink density, which are essential for preserving mesogenic order during polymerization [68]. Photoinitiators must remain compatible with the nematic phase to avoid disruption of alignment, since mislocalized radical formation can generate domain defects or thermal stresses that diminish final anisotropy [51]. Free radical photoinitiators such as Irgacure 651, Irgacure 369, and TPO are widely used because they efficiently generate radicals in relatively viscous oligomeric precursors [6, 67]. These initiators support rapid network propagation and allow uniform conversion in side chain LCEs formulations based on RM82 and related diacrylates [67]. Comparative optical studies show that photoinitiators with deeper penetration profiles reduce crosslink gradients in bulk samples and therefore lead to higher macroscopic actuation fidelity [51]. In fiber fabrication platforms, consistent curing of sub‐millimeter strands has been demonstrated using Irgacure‐based systems, which preserve alignment along the length of the drawn filament.

### 2.5 Inhibitor

Inhibitors are essential additives used to stabilize reactive monomers and oligomers during liquid crystal elastomer precursor formulation, storage, and processing. Their primary function is to suppress premature polymerization, particularly in acrylate and methacrylate systems that are prone to spontaneous radical generation from heat, light, or trace impurities [69]. Common inhibitors such as hydroquinone, hydroquinone monomethyl ether (MEHQ), and butylated hydroxytoluene (BHT) act as radical scavengers that temporarily neutralize initiating species and extend the workable pot life of the reactive mixture [67].

In thiol acrylate Michael addition formulations, inhibitors also help regulate early‐stage oligomer growth by modulating competing radical pathways [70]. Low concentrations, typically 50–500 ppm, of MEHQ or BHT maintain stability during processing steps such as mixing, degassing, extrusion, and alignment without significantly slowing UV‐initiated curing [67]. Proper inhibitor loading ensures that viscosity remains predictable during prepolymer formation, enabling reproducible filament extrusion or direct ink writing. Insufficient inhibition may lead to premature gelation, nozzle clogging, or disrupted mesogen alignment, whereas excessive inhibition can hinder complete curing and reduce final network integrity. The inhibitor plays a vital role in maintaining formulation stability, preserving processing windows, and ensuring uniform crosslinking once polymerization is deliberately initiated, thereby improving the reproducibility and mechanical quality of liquid crystal elastomer actuators and printed structures [52, 71].

### 2.6 Additives/Fillers

Additives encompass a broad class of auxiliary materials incorporated into liquid crystal elastomer formulations to modify processing behavior, enhance stability, and tailor the final functional properties of the elastomer network. While not directly participating in crosslinking or mesogen alignment, additives play critical supporting roles that influence printability, optical performance, actuator durability, and long‐term environmental resistance [6]. Processing additives, including plasticizers, rheology modifiers, and surfactants, are used to tune viscosity and flow behavior during extrusion, filament drawing, or direct ink writing [20]. Plasticizers such as phthalate‐free oligomeric modifiers or reactive diluents reduce intermolecular interactions within the prepolymer, enabling smoother deposition and improved layer fusion without disrupting liquid crystalline phase behavior when used at low concentrations [67]. Surfactants and wetting agents help maintain uniform dispersion of pigments, nanoparticles, or dyes introduced for optical functionality [72]. Functional additives introduce additional capabilities to the liquid crystal elastomer network. UV absorbers and hindered amine light stabilizers improve photostability during prolonged actuation or outdoor exposure [73]. Antioxidants such as BHT or phosphite stabilizers mitigate thermal degradation during repeated heating and cooling cycles [74]. Incorporation of conductive fillers, for example, carbon black, carbon nanotubes, or silver flakes, magnetic nanoparticles, or luminescent dyes, enables multimodal actuation, sensing, or photonic responses without fundamentally altering the liquid crystalline phase, provided dispersion is well controlled [75]. Overall, the presence and selection of additives significantly affect the reliability, printability, and long‐term functional stability of liquid crystal elastomers. Proper formulation ensures that these components enhance performance without compromising mesogen alignment, mechanical anisotropy, or actuation efficiency [52, 71].

## 3 Prepolymer Conditioning

### 3.1 Processing Parameters

To enhance clarity and facilitate comparison across different studies, key processing parameters discussed in this review are defined in a consistent manner. The processing window refers to the range of temperature, viscosity, and curing conditions within which a LCE precursor can be effectively processed into a stable filament while preserving mesogen alignment prior to crosslinking. The viscosity window denotes the range of viscosities that enables smooth extrusion along with sufficient post‐deposition shape retention. Furthermore, yield stress represents the minimum stress required to initiate flow, thereby determining whether the material behaves as a solid‐like or liquid‐like system during processing. Finally, alignment retention describes the ability of shear‐induced mesogen orientation to persist during and after processing until the network is fully fixed. These definitions provide a foundational framework applied throughout this review to ensure consistency in the discussion of LCEs filament fabrication.

### 3.2 Rheology Tuning

**FIGURE 4:**
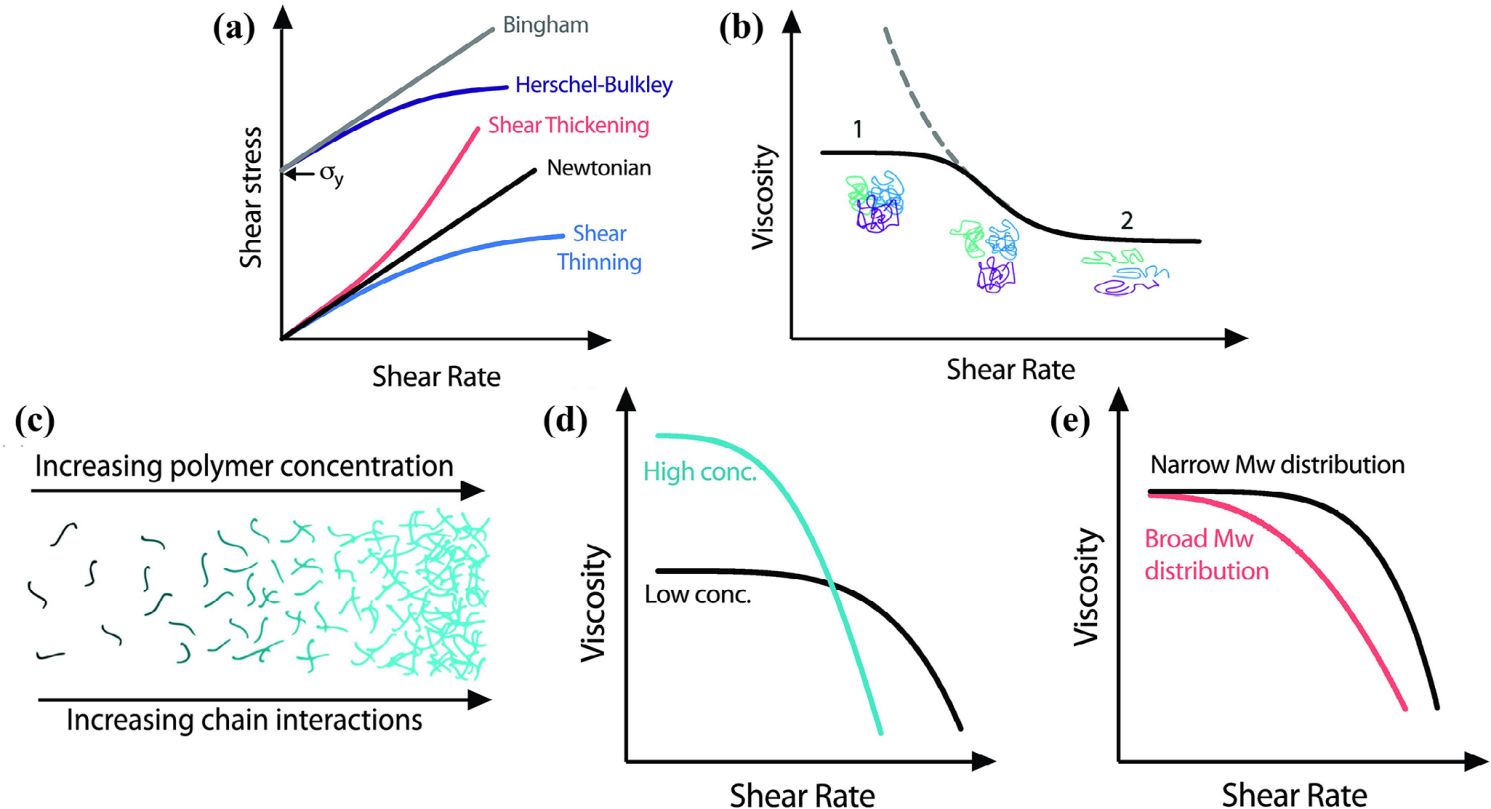
(a) Flow curves showing Newtonian, shear‐thinning, shear‐thickening, Bingham, and Herschel–Bulkley behaviors, with *σ*ᵧ indicating the yield stress. (b) Shear‐thinning viscosity profile showing low‐shear Newtonian plateau (1) and high‐shear Newtonian plateau (2); the dashed line indicates the behavior of a yield‐stress material. (c) Effects of the polymer concentration on chain interactions and (d) viscosity with respect to the shear rate. (e) Effects of polydispersity on shear‐thinning behavior. Reproduced from Cooke & Rosenzweig [77], CC by 4.0.

Polymer solutions consist of solvated chains whose interactions depend strongly on polymer concentration and molecular‐weight distribution (Figure 4a). In dilute systems, chain–chain interactions are minimal, whereas increasing concentration leads to chain overlap, noncovalent interactions, and ultimately entanglement [76, 77]. Greater entanglement increases viscosity, elevates the zero‐shear plateau, and decreases the critical shear rate at which shear thinning begins [19, 20, 77]. At very high concentrations, the critical shear rate becomes indistinct, resulting in an immediate transition into shear thinning with a more rapid viscosity decay (Figure 4b). Molecular weight (*Mw*) and its distribution further influence rheological behavior. Systems with broad Mw distributions exhibit weaker shear‐thinning behavior and a less distinct critical shear rate than those with narrow distributions (Figure 4c,e). Such distributions may arise through blending polymer batches with different *Mw* values or intentionally mixing heterogeneous polymers [19, 20, 76, 77].

Rheology plays a central role in defining how LCE precursors deform under stress, thereby determining filament processability and director‐alignment fidelity during manufacturing [78]. In filament‐based LCEs fabrication, including direct‐ink writing, fused‐filament deposition, and continuous extrusion, rheological control governs strand integrity, surface quality, and the preservation of flow‐induced mesogen orientation prior to crosslinking [33, 37]. As illustrated in Figure 4, LCEs precursors rarely behave as Newtonian fluids; instead, they display shear‐thinning, shear‐thickening, Bingham, or Herschel–Bulkley responses linked to intermolecular entanglement, mesogen ordering, and early‐stage network formation. Shear‐thinning behavior is particularly beneficial during extrusion [76, 77]. It can be observed from Figure 4d that reduced viscosity at elevated shear rates lowers pressure requirements and stabilizes deposition [79]. Many LCE oligomers and gels also exhibit yield‐stress behavior, generating a gel‐like elastic response that prevents filament spreading, with viscosity‐shear‐rate curves transitioning from a near‐zero‐shear plateau to a high‐shear regime or, in yield‐stress systems, lacking a true plateau due to suppressed flow below the critical stress [80].

The rheological properties of LCE precursors can be tuned through molecular‐weight control, partial Michael‐addition prepolymerization, thermal conditioning, or incorporation of microgels, fumed silica, or cellulose nanocrystals [42, 52, 81]. Such tuning enables uniform filament formation during continuous fiber drawing while preserving shear‐induced alignment over extended lengths. Similar control is required in wet‐spun polydomain LCE fibers, where optimized precursor rheology prevents coagulation‐induced collapse and supports post‐processing actuation modes. In woven LCE yarns, maintaining rheological stability during fiber formation is essential for preserving programmable deformation pathways after textile integration. Ultimately, rheology defines the processing window in which LCE precursors must simultaneously flow, self‐support, and maintain liquid‐crystalline order. Precise control ensures robust formation of anisotropic filaments and printed architectures, allowing shear‐aligned mesogens to be retained and locked during curing conditions critical for reproducible actuation in filamentary LCE systems [7, 48, 77].

### 3.3 Viscosity Control

Viscosity is a critical parameter governing extrusion behavior, filament stability, and alignment retention in LCE filament fabrication. Although a wide viscosity range has been reported, different regimes lead to fundamentally distinct processing outcomes. At low viscosities (<50 Pa s), precursors exhibit excellent flowability but suffer from poor shape retention and rapid relaxation of shear‐induced alignment, resulting in filament spreading and reduced dimensional fidelity [6, 17, 82]. In contrast, intermediate viscosities (50–500 Pa s) provide an optimal balance between flow and viscoelastic recovery, enabling stable extrusion, uniform filament formation, and effective preservation of mesogen alignment. At high viscosities (>500 Pa s), enhanced entanglement improves shape retention and alignment stability; however, the required high extrusion pressures may introduce flow instabilities, discontinuities [83, 84], and reduced molecular mobility, limiting efficient alignment. These differences demonstrate that, while multiple viscosity ranges are processable, filament quality and actuation performance are maximized within a narrow intermediate viscosity window. Importantly, this window is system‐dependent and must be co‐optimized with shear conditions and curing kinetics to ensure that flow‐induced alignment is effectively retained and fixed during processing. Viscosity directly influences extrusion smoothness, filament continuity, and the dimensional stability of deposited features during LCE filament fabrication [37, 42] (Table 3).

**TABLE 3:** Viscosity ranges and processing conditions for representative LCES precursor chemistries.

LCES chemistry	Typical viscosity range (Pa s)	Processing temperature	Relevance to printability	Refs.
Thiol‐acrylate (Michael‐addition)	50–2000 Pa s	Room temperature‐60°C	Viscosity readily tuned via oligomerization time; strong shear‐thinning; widely used for DIW.	[33, 52, 70]
Hydrosilylation‐based LCES oligomers	200–5000 Pa s	40°C–90°C	Higher base viscosity; often requires heating for stable extrusion; excellent filament integrity.	[32, 81, 85]
Acrylate (photocurable) mesogen oligomers	10–500 Pa s	Room temperature	Low viscosity; may require rheology modifiers to prevent spreading; fast UV‐curing response.	[6, 17, 82]
Siloxane‐rich polysiloxane LCES inks	500–10 000 Pa s	25°C–70°C	High viscosity and elastic character; suitable for extrusion and magnetically assisted alignment.	[83, 84]
Nematic‐phase LCES oligomers (temperature dependent)	30–1000 Pa s	Near LC‐Iso transition	Viscosity strongly phase‐dependent; requires precise thermal regulation for printing and alignment.	[1, 31]

In thiol‐acrylate systems, viscosity is commonly regulated through controlled oligomerization during the Michael‐addition stage. Incremental chain extension increases molecular weight and intermolecular entanglement, improving the self‐supporting character of the deposited filament while maintaining printability [70, 86]. Catalyst concentration provides an additional lever, where weak nucleophilic bases such as dipropylamine and triethylamine enable a gradual viscosity rise, whereas stronger bases accelerate network formation and narrow the usable processing window [52]. This strategy has been successfully employed in multiple LCEs manufacturing approaches, including direct ink writing, continuous filament drawing, and hybrid wet‐spinning, where rheological stabilization is essential to avoid strand collapse and inconsistent crosslinking [17, 33, 49]. Temperature plays a dominant role in viscosity tuning, particularly for mesogen‐rich oligomers whose ordering state directly affects mobility. Approaching the nematic‐isotropic transition temperature reduces viscosity by disrupting positional and orientational constraints within the mesogenic phase, enabling smoother nozzle flow and enhanced surface uniformity [1, 80]. However, excessive thermal thinning risks partial loss of director orientation, resulting in reduced actuation fidelity after curing. Continuous fiber manufacturing of LCEs actuators demonstrates this trade‐off: elevated processing temperatures facilitate extrusion, but alignment retention and contraction performance are preserved only when ordering is fixed immediately after viscous flow. Similarly, wet‐spinning routes utilize viscosity windows that prevent droplet coalescence and coagulation instability, ensuring stable filament formation prior to post‐treatment or crosslinking.

Beyond molecular weight and temperature, viscosity can be modulated through formulation chemistry. Reactive diluents such as monoacrylates reduce intermolecular friction and lower ink viscosity without compromising the final network. Nanofillers such as fumed silica, cellulose nanocrystals, or microgel additives introduce reversible physical entanglement, leading to an increase in low‐shear viscosity and enhancing filament shape retention [37, 42, 81]. These strategies have proven particularly effective in LCE textile fiber systems, where stable viscosity profiles prevent spreading during deposition and maintain local ordering that is later encoded into woven or embroidered actuating structures. Viscosity establishes the functional processing window in which LCEs precursors remain extrudable, self‐supporting, and capable of retaining shear‐induced molecular alignment until crosslinking. Maintaining viscosity within this window is essential for defect‐free filament formation, reproducible mesogen orientation, and reliable actuation performance in continuous fibers, printed lattices, and textile‐integrated LCE systems [6, 7, 48].

### 3.4 Interplay Between Rheological and Processing Parameters

The intricate relationship between viscosity, extrusion pressure, and flow rate constitutes a coupled system that ultimately dictates the quality of the LCE filament. These parameters are not independent; rather, they exist in a state of non‐linear interdependence primarily governed by the power–law fluid behavior of LCEs prepolymers. For instance, as a prepolymer is conditioned toward a higher viscosity to enhance shape retention, the required extrusion pressure must be increased to maintain a constant flow rate. However, this elevation in pressure can induce “die swell” or flow instabilities that jeopardize the internal mesogen alignment. Similarly, the flow rate directly influences the shear rate within the nozzle, which is the primary driver for shear‐induced alignment. While higher flow rates can enhance orientation, they simultaneously reduce the residence time available for the mesogens to stabilize, potentially leading to “melt fracture” if the stresses exceed the material's cohesive limits. Furthermore, because LCE rheology is highly sensitive to thermal fluctuations, minor changes in processing temperature can shift the viscosity window, triggering a cascade of necessary adjustments in pressure and speed to prevent non‐uniformities. Consequently, achieving a stable processing window requires a holistic optimization strategy that accounts for these coupling effects to ensure that maximum shear‐induced orientation is achieved without compromising the structural integrity or dimensional precision of the extrudate (Table 3).

### 3.5 Shear‐Thinning Behavior

Shear‐thinning is one of the most advantageous rheological characteristics for extrusion‐based LCEs fabrication, especially in direct‐ink writing (DIW). Under increasing shear rate, the viscosity of the precursor decreases as polymer chains and mesogenic domains orient along the flow direction, lowering hydrodynamic resistance and facilitating passage through micron‐scale nozzle geometries [79, 87]. This transient ordering allows flow under pressure while preserving structural integrity at rest, enabling stable deposition and improved filament shape retention after extrusion [81]. Importantly, the recovery of high viscosity at low shear acts as a natural self‐support mechanism, suppressing collapse and providing a pathway to encode mesogen orientation directly during printing [1, 7].

Thiol‐acrylate LCE oligomers typically exhibit strong shear‐thinning due to their intermediate molecular weight distributions and partially formed network motifs, which promote chain disentanglement under flow [52, 70]. Formulations containing rheology modifiers such as fumed silica, cellulose nanocrystals, or microgels further enhance pseudoplasticity by introducing reversible microstructures that break down under shear and reform after deposition [42, 81, 88]. This behavior is evident in continuous filament drawing systems, where shear‐induced ordering enables alignment locking across meter‐scale fibers and directly translates into reversible strain upon actuation. Similarly, shear‐thinning facilitates stable wet‐spinning of polydomain LCE yarns, preventing jet breakup and enabling uniform filament formation prior to final crosslinking. The magnitude of shear‐thinning influences downstream manufacturing metrics including extrusion pressure, dimensional fidelity, and achievable print resolution [89, 90]. Stronger shear‐thinning reduces required printing pressure and mitigates nozzle pulsation, while insufficient shear response increases filament swelling, bead formation, and susceptibility to shear‐banding during deposition. In filamentary LCEs actuators and textile‐integrated yarns, optimizing this response is essential to ensure that molecular alignment programmed during flow remains preserved through the curing step, ultimately determining actuation efficiency, durability, and thermal cycling stability [6, 17, 42].

### 3.6 Yield‐Stress Tuning

Yield stress defines the minimum applied stress required to initiate flow, distinguishing viscoelastic solids from true fluids and establishing a key rheological threshold for extrusion‐based LCEs fabrication. In practical terms, a printable LCE formulation must maintain a moderate yield stress: below this threshold, the precursor behaves as a weak viscoelastic liquid, spreading after deposition and losing dimensional fidelity; above it, excessive resistance to deformation impedes extrusion, increases nozzle pressure, and promotes irregular flow regimes [91, 92, 93]. In filaments and printed lattices, an appropriately tuned yield stress enables self‐supporting deposition and mitigates gravitational sag, especially in overhang geometries or multi‐layer architectures where filament integrity must be sustained prior to curing [37, 89]. Yield‐stress tuning in LCE systems is commonly achieved through molecular and microstructural strategies. Controlled Michael‐addition oligomerization increases partial network formation, enhancing intermolecular entanglement and raising low‐shear resistance without fully compromising flow [52, 70]. Incorporation of thixotropic particles such as fumed silica, cellulose nanofibrils, nanoclays, or microgel agents introduces reversible weak particle networks that collapse under shear and reform at rest, preventing post‐extrusion spreading and supporting vertical build heights [94, 95, 96]. These approaches are especially effective in DIW‐printed LCE structures, where successful printing of unsupported or architected geometries relies on the balance between shear‐induced flowability and solid‐like recovery after deposition [42, 48].

**FIGURE 5:**
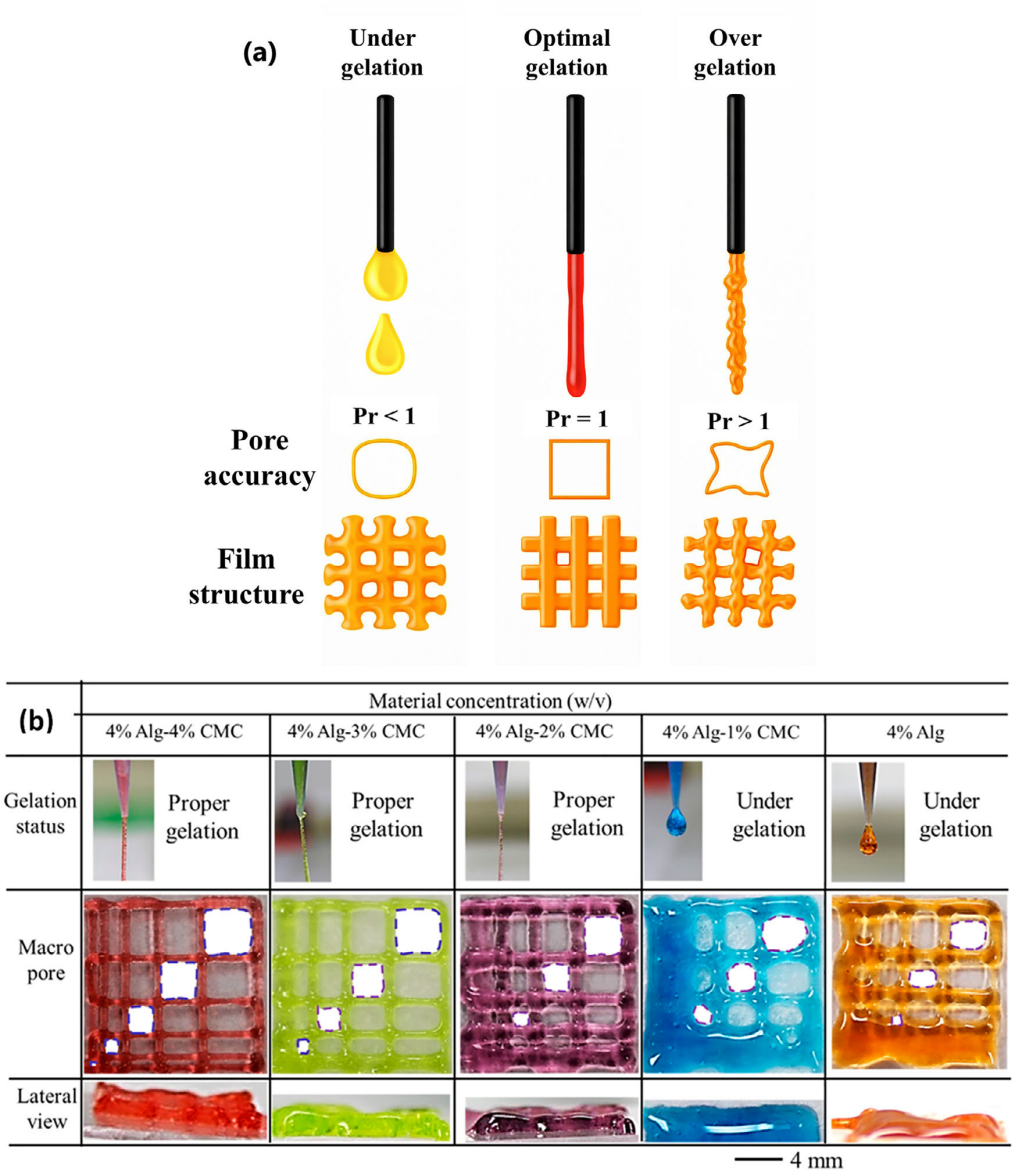
Effect of polymer concentration on filament fusion and spreading behavior. Higher CMC concentration increases ink cohesion and stiffness, reducing filament swelling and coalescence after deposition. (a) Under gelation, optimal gelation, and over gelation(b) Filament fusion test showing the effect of carboxymethyl cellulose (CMC) concentration on bioink extrusion behavior. Higher CMC concentrations increase polymer entanglements, resulting in stiffer extrusion, reduced filament swelling, and less coalescence of printed fibers compared to lower or 0% CMC. Reproduced from Habib et al., Materials 11 (2018), CC BY 4.0. [77]).

Fiber‐based LCEs manufacturing provides further evidence of yield‐stress control as a central design parameter. In continuous filament extrusion, sufficient yield stress prevents strand collapse as shear conditions change from internal nozzle flow to ambient post‐deposition relaxation, enabling alignment retention along meter‐scale fiber lengths. Wet‐spinning systems exhibit similar behavior: inks with insufficient resting stiffness undergo rapid swelling or jet breakup in coagulation baths, while tuned yield‐stress precursors maintain cylindrical geometry and permit controlled post‐processing of twist, contraction, or heterogeneous phase structures. Textile‐integrated LCE yarns further demonstrate that yield‐stress tuning is critical for preserving programmed anisotropy during weaving and embroidery, preventing filament flattening under contact stresses and stabilizing deformation pathways encoded during drawing. Collectively, these observations highlight yield stress as an enabling parameter rather than a passive material characteristic. Figure 5a,b shows that optimization determines whether an LCE precursor transitions smoothly from a flowing state under pressure to a dimensionally stable solid capable of preserving alignment until crosslinking. Formulations calibrated to this edge demonstrate improved filament fidelity, reduced structural defects, and consistent actuation behavior across continuous fibers, DIW‐printed devices, and textile‐embedded LCEs architectures [37, 81].

### 3.7 Phase‐State Control

Phase‐state control refers to regulating whether the LCE precursor is processed in the nematic, paranematic, or isotropic state, a factor that strongly influences viscosity, molecular mobility, flow‐induced director alignment, and ultimately the actuation performance of the printed elastomer [97]. Phase‐state selection during processing not only governs flow behavior but also determines how effectively shear‐induced molecular orientation can be retained and later locked into the elastomer network [98]. The nematic phase typically exhibits anisotropic viscosity and higher orientational order, enabling efficient transfer of shear‐induced alignment into the final network, whereas the isotropic phase provides lower viscosity and improved flow uniformity at the expense of mesogen ordering [1, 45, 48]. Maintaining a nematic or paranematic state during extrusion is advantageous for imprinting the desired director orientation directly into the printed filament; however, some DIW and nozzle‐based processes deliberately operate above the isotropic‐nematic transition temperature to minimize viscosity and eliminate trapped air, followed by controlled cooling, alignment, and crosslinking to recover anisotropy [6, 7, 42]. Practical phase‐state tuning is achieved through temperature‐controlled print beds, heated syringes, resistive or infrared localized heating, and feedback‐controlled thermal gradients that stabilize the precursor's rheological window during deposition [37, 81]. Precise regulation of phase state during printing is therefore essential for ensuring defect‐free extrusion, reproducible microstructure, and robust actuation behavior in 4D‐printed LCEs architectures [41].

### 3.8 Shear and Velocity Profiles Inside Nozzles

Under pressure‐driven capillary flow, LCEs precursor inks experience non‐uniform velocity and shear‐rate distributions across the nozzle radius. These internal gradients govern the apparent viscosity of the formulation, the stability of flow, and the extent to which shear aligns mesogens prior to deposition [79, 99]. Such flow‐induced alignment during extrusion has been exploited in additive manufacturing strategies to program anisotropic deformation and shape morphing in soft materials [100]. In Newtonian fluids, the axial velocity profile follows a classical Poiseuille parabola, characterized by a maximum centerline velocity and a linear increase in shear rate toward the nozzle wall. LCEs inks deviate from this ideal behavior due to their strongly non‐Newtonian character: shear‐thinning formulations develop flattened or blunted velocity profiles as viscosity decreases at high shear, reducing the wall‐center line contrast and suppressing radial velocity gradients [77, 79, 80, 81, 82, 83, 84, 85, 86, 87, 88, 89, 90, 91, 92, 93, 94, 95, 98, 99, 101, 102].

For yield‐stress or Herschel–Bulkley‐type LCEs inks, the flow field may include a central plug‐flow zone in which the local stress remains below the yield stress, surrounded by a high‐shear annulus adjacent to the walls [103, 104]. The plug radius expands with increasing yield stress or decreasing pressure gradient, altering filament diameter and surface uniformity during extrusion. While plug‐flow reduces internal velocity gradients, it can induce extrusion pulsation, surface roughening, and intermittent flow effects that are particularly evident in partially crosslinked or highly elastic formulations [37, 105]. Such flow behaviors have been observed in continuous LCE fiber manufacturing, where plug‐like extrusion promotes filament stability but must be balanced against alignment retention to avoid mechanical defects in long fiber bundles.

### 3.9 Controlled Partial Crosslinking

Controlled partial crosslinking is a key strategy for tuning the printability and structural retention of LCE precursor inks. By initiating only a fraction of the available reactive groups, the precursor transitions from a low‐viscosity oligomer to a lightly crosslinked, gel‐like network that resists spreading yet remains extrudable [37, 81]. Unlike full polymerization, which locks mesogenic domains and terminates alignment, partial crosslinking increases elastic storage without eliminating flowability, enabling stable deposition at moderate extrusion pressures and preservation of flow‐induced director orientation [33, 70]. This intermediate viscoelastic state can be achieved through several mechanisms. Reducing photoinitiator loading or UV dose limits radical generation and slows propagation, while weak tertiary‐amine catalysts delay Michael‐addition kinetics and extend the prepolymer processing window [52, 67]. Thermal pre‐curing at controlled temperatures similarly promotes short‐range network formation without reaching percolation thresholds that hinder flow. Such staged‐cure strategies have proven effective in direct‐ink writing and extrusion of programmable LCES architectures, where printed filaments must retain geometry over multi‐layer assemblies prior to final curing [42, 81]. Fiber manufacturing provides compelling evidence of the role of partial crosslinking in stabilizing alignment. During continuous filament drawing, low‐dose UV exposure or catalyst‐limited oligomerization allows shear‐aligned mesogens to be partially immobilized before final curing, preventing recoil and enabling uniform actuation performance across meter‐scale fibers. In wet‐spun LCE systems, similar controlled curing prevents interfacial swelling in coagulation baths and stabilizes cylindrical filament geometry prior to full network formation. Textile‐integrated LCE yarns also benefit from staged‐cure protocols, which maintain fiber stiffness during weaving or embroidery, limiting filament flattening and preserving embedded deformation pathways.

Following deposition or drawing, full crosslinking is typically effected via high‐intensity UV irradiation or thermal cure and locks the nematic director orientation into a permanent elastomeric network. This final cure step determines the material modulus, the magnitude of spontaneous strain, and the durability of thermal or optical actuation cycles [6, 17, 32]. When staged appropriately, partial and full crosslinking together define a processing window that preserves printability, maintains geometric fidelity, and stabilizes flow‐programmed anisotropy, enabling reliable actuation in printed LCE devices as well as filament‐based actuator textiles [6, 17, 37, 42].

## 4 Filament Formation and Deposition Mechanics

### 4.1 Processing Routes (Printing/Spinning/Jetting Modes)

**FIGURE 6:**
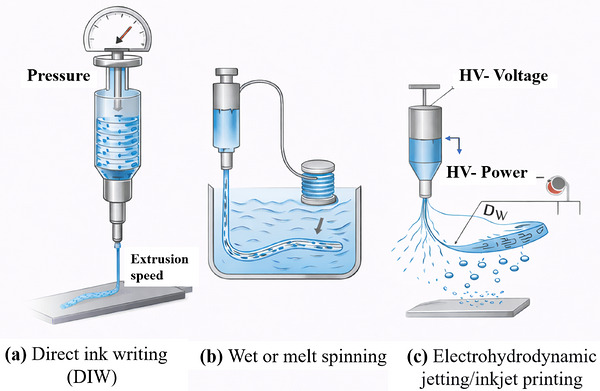
Filament production pathways for LCEs fabrication. Comparison of three major filament‐forming techniques: (a) DIW extrusion, where pressure‐driven flow deposits shear‐aligned filaments; (b) wet or melt spinning, in which polymer jets are drawn and solidified through coagulation or thermal cooling; and (c) electrohydrodynamic jetting/inkjet printing, producing ultrafine fibers or droplets under electric‐field‐driven jet break‐up.

LCE filaments can be produced through pressure‐driven extrusion, spinning‐based draw processes, or electrohydrodynamic jetting, as illustrated in Figure 6. These pathways differ in how they impose shear, extension, and phase transitions, and therefore produce distinct director fields and actuation behaviors [1, 7, 40]. In DIW extrusion (Figure 6a), highly viscous LCE precursor inks are extruded through a rigid nozzle under pneumatic or mechanical pressure. The shear‐gradient distribution inside the nozzle governs filament continuity, interlayer bonding, and defect formation. Shear‐induced director alignment is retained when rheology, nozzle geometry, and post‐extrusion solidification are correctly staged [80, 102, 106].

Alignment efficiency is often interpreted using the Weissenberg number (*Wi* = *λγ̇*) or the Deborah number (De), which compares the relaxation time of the liquid‐crystal network with the imposed shear rate; values *Wi* > 1 indicate that flow deformation dominates over molecular relaxation, enabling persistent director alignment during extrusion. This modality supports architected 4D structures but relies on carefully tuned viscosity and yield stress to limit filament sagging [79, 107]. Spinning methods (Figure 6b) introduce controlled extensional flow and draw ratios absent in DIW. Wet‐spinning and melt‐spinning apply axial stretching that produces uniform orientation across the filament cross‐section and large reversible actuation strains [32, 85]. Electrohydrodynamic jetting and inkjet‐based approaches (Figure 6c) use electric‐field‐driven jet breakup to generate ultrafine fibers or droplets, enabling fine spatial control but typically requiring lower viscosities and careful charge stabilization. In modern approaches, continuous drawing has enabled meter‐scale filaments whose alignment is locked during in situ curing, enabling textile‐level integration.

### 4.2 Nozzle Design

Nozzle or spinneret design is central to controlling filament geometry, internal shear fields, and, for LCEs, director alignment during printing. In DIW, the nozzle acts as a converging capillary in which pressure‐driven flow generates a shear‐rate profile that depends on the ink rheology and the nozzle geometry (length, taper, and internal diameter). Analytical and numerical studies show that the shear history inside the nozzle governs both the apparent viscosity at the exit and the magnitude and uniformity of the shear stress applied to cells, nanoparticles, or mesogens [80, 108, 109]. For LCE inks, flow‐induced alignment can be enhanced by tuning extrusion parameters and nozzle geometry. Gradually converging channels, tapered dies, and long‐shear sections have been shown to increase molecular orientation and reduce director defects compared with abrupt transitions, due to more uniform velocity gradients and stable extensional flow regimes [17, 41, 48]. Similar considerations apply to spinning and jetting spinnerets, where the draw ratio and outlet shape control filament diameter and orientation. As a result, nozzle/spinneret design is increasingly treated as a co‐design variable together with ink rheology and printing path when targeting programmed actuation in 3D‐ and 4D‐printed LCEs systems [81, 101, 110].

#### 4.2.1 Inner Diameter Selection

The inner diameter (ID) selection defines the compromise between printing resolution, required extrusion pressure, shear rate, and risk of clogging. For a power–law ink flowing through a cylindrical nozzle, the wall shear rate scales inversely with the cube of the radius (*γ̇*_w ∝ *Q*/*R* ^3^), so decreasing ID enhances shear‐thinning and alignment but rapidly increases pressure drop and shear stress on embedded inclusions such as cells or fibers [80, 111]. Systematic printability studies suggest that typical DIW and bioprinting nozzles operate in the 100–410 µm range for hydrogel‐like or LCEs inks, balancing filament diameters suitable for architected lattices with acceptable extrusion pressures and cell viability where relevant [112, 113]. Smaller diameters improve feature resolution but intensify shear‐induced damage and clogging risk in particle‐laden or partially crosslinked inks, while larger diameters ease flow but promote filament sagging and loss of geometric fidelity [91, 109, 114]. In LCEs DIW, ID also influences mesogen orientation and actuation anisotropy: narrower nozzles and higher shear rates induce stronger flow alignment but can over‐orient or disrupt texture if combined with excessively high extrusion velocities [106, 110]. Recent work on printability maps and resolution models further shows that nozzle diameter interacts with nozzle height and printing speed to define a domain of stable filament formation with minimal defects, offering design guidelines for high‐flatness layers and complex LCEs architectures [115, 116].

#### 4.2.2 Coaxial Nozzles

In bioprinting, coaxial extrusion has been widely used to produce hollow hydrogel filaments and endothelialized channels [117]. It also enables the fabrication of constructs with mechanically supportive shells and cell‐laden cores [118]. These capabilities have dramatically expanded the accessible design space of extrusion‐based printing [117, 118]. Recent work has adapted coaxial DIW to fabricate core–shell metallic lines, coaxial electrostatic fibers, and multicore transmission lines, demonstrating controlled core diameters, tunable shell thicknesses, and complex electrical or mechanical functionalities within a single filament [119, 120, 121]. For LCEs, coaxial printing can be used to embed reinforcing fibers, conductive cores, or stimuli‐responsive domains inside an actuating shell, or conversely to encapsulate an LCE's core within a protective or adhesive outer layer [122, 123]. Emerging studies on coaxial DIW of cholesteric liquid crystals and polysaccharide‐based cores illustrate the potential for simultaneously programming optical and mechanical responses [124]. Key design parameters include the ratio of inner to outer diameters, relative flow rates of core and shell inks, and interfacial compatibility, all of which influence core stability, shell integrity, and the ability to maintain alignment and crosslinking conditions in the LCEs phase [102, 117].

#### 4.2.3 Taper Geometry and Contraction Ratio

Shear profile optimization aims to control the spatial distribution of shear stress and shear rate within the nozzle and at the exit, ensuring filaments are produced with uniform geometry and desired microstructural alignment while minimizing defects or damage. For non‐Newtonian DIW inks, both analytical and computational models show that shear stress varies strongly across the nozzle radius. By carefully tailoring nozzle length, taper angle, and contraction ratio, the shear field can be redistributed to smooth gradients, preventing non‐uniform director orientation and localized overstressing [102, 108, 110, 125]. Hyperbolic and smoothly converging nozzle profiles, for instance, minimize extensional stress concentrations and promote more uniform director alignment compared with abrupt conical geometries, directly improving actuation uniformity [110, 125]. In bioprinting, optimization of shear profiles is closely linked to cell viability: narrower contraction angles and longer taper lengths reduce peak shear stresses and enhance post‐printing survival [126, 127]. At the macroscopic scale, shear profile control is also achieved by adjusting extrusion pressure, printing speed, and nozzle height to maintain stable, laminar flow and consistent filament diameter. Printability maps derived from experiments and simulations identify combinations of yield stress, shear‐thinning index, and process parameters that produce defect‐free filaments with minimal beading or breakage [102, 128]. For LCEs, shear profile optimization is particularly critical because the same shear that shapes the filament also aligns mesogens; careful tuning allows decoupling of alignment strength from detrimental effects such as shear banding or premature crosslinking, enabling spatially programmed actuation in printed structures [101, 106, 125, 129].

### 4.3 Thermal and Phase Control During Deposition

Thermal control during deposition governs flow stability, liquid crystalline phase state, and filament morphology in LCEs' extrusion, spinning, and wet‐spinning processes. Across LCEs, fiber manufacturing and smart‐textile integration, nozzle temperature, reservoir heating, and phase regulation have been shown to directly influence process fidelity and final actuator performance [37, 80]. In direct ink writing and melt extrusion, elevating nozzle temperature reduces viscosity, mitigates pulsatile flow, and delays premature crosslinking in thiol‐acrylate and hydrosilylation‐based systems [37, 80]. Comparable thermal strategies are used in wet‐spinning to suppress early gel‐front formation and maintain stable draw ratios. Continuous fiber drawing at elevated temperatures produces uniform filaments and improves drawability, resulting in high‐aspect‐ratio actuating fibers with strong reversible strains [129, 130].

### 4.4 Flow‐Induced Alignment

Flow‐induced alignment is the primary mechanism through which LCEs precursor inks develop anisotropic microstructure during extrusion, spinning, or jetting. As the precursor travels through a nozzle or spinneret, mesogenic domains experience shear and extensional stresses that bias their directors toward the flow direction. The resulting orientational order governs the macroscopic functionality of the final network, including actuation direction, achievable reversible strain, optical birefringence, and mechanical anisotropy [5, 6, 7, 9, 48]. Recent studies further demonstrate that flow alignment controls not only deformation magnitude but also deformation pathways, enabling programmable bending, twisting, and ribbon‐like shape morphing in nematic and cholesteric LCEs [131, 132]. At larger scales, controlled alignment under extensional flow has been leveraged to produce continuously drawn fibers and 4D‐engineered yarns with persistent contractile actuation, as exemplified by continuous melt‐drawing platforms such as FibeRobo [18] and by shape‐programming methodologies that translate microscale director fields into mesoscale morphing responses [9, 129].

#### 4.4.1 Shear‐Induced Director Alignment

Under pressure‐driven capillary flow, radial shear gradients develop across the nozzle cross‐section: wall regions experience maximum shear, while the core remains minimally deformed [79, 99]. In nematic or paranematic states, this gradient rotates mesogens into the axial direction, generating a uniaxial director field that can be preserved through curing [5, 48]. Shear‐based alignment has been repeatedly demonstrated in DIW printing, wet‐spinning, and melt extrusion, where uniform director orientation enhances actuation strain and stiffness anisotropy [40, 42, 77]. Antigelation spinning confirms that sustained shear during extrusion stabilizes jets and suppresses defect formation before gelation, preventing premature coagulation and breakup [133]. Comparable shear alignment is observed in coaxially extruded core–shell LCES fibers, where controlled solvent exchange yields smooth surfaces and uniform diameter [58, 59]. At the textile scale, shear‐aligned wet‐spun LCES yarns enable weaving and embroidery while retaining meter‐scale continuity [22, 129, 130].

#### 4.4.2 Extensional‐Flow Alignment

As filaments exit the nozzle, the flow transitions from predominantly shear to predominantly extensional. Extensional stresses stretch the polymer network along the axial direction and typically produce more uniform alignment than shear alone, reducing radial director gradients [76, 134, 135]. Alignment strength scales with the draw ratio, defined as the take‐up velocity divided by the extrusion velocity. High draw ratios thin the filament and amplify alignment, producing fibers with significantly enhanced actuation performance [20, 41, 45]. Extensional alignment dominates melt‐spinning and dry‐jet wet‐spinning, enabling tight microstructural control in sub‐micron domains [88, 89]. Similar principles govern braided and woven LCE filaments, where take‐up velocity defines bending modes and torsional response [18, 45, 57, 129]. Recent advancements in fiber fabrication have significantly pushed the limits of mechanical performance and actuation strain. Notably, Lv and co‐workers [136] developed a series of high‐performance LCES fibers through optimized molecular design and processing. These fibers exhibit exceptional mechanical robustness and high work density, bridging the gap between laboratory‐scale filaments and industrial‐grade functional yarns suitable for high‐load soft robotic applications.

#### 4.4.3 Competition Between Flow Orientation and Relaxation

Flow‐induced orientation competes with relaxation processes in which mesogens return toward equilibrium. Persistence depends on the ratio between flow‐orientation time (*τ*_flow) and molecular relaxation time (*τ*_relax). Alignment is retained when *τ*_flow < *τ*_relax but decays when relaxation is faster than orientation [5, 6, 9]. This competition governs jet stability in extrusion, fiber formation in wet‐spinning, and microstructural development in DIW [48, 98]. Temperature strongly modulates *τ*_relax. Near or above the isotropic transition temperature, relaxation accelerates, reducing director fidelity and actuation performance [45, 48]. Similar decay has been reported in 4D‐printed LCE microarchitectures, where elevated processing temperatures destroy programmed texture [14, 64]. To prevent relaxation‐driven disorder, rapid post‐extrusion curing, solvent‐induced gelation, or partial pre‐crosslinking are used to immobilize aligned mesogens before orientation decays [37, 42, 81].

### 4.5 In‐Process Solidification/Gel Fixation

In‐process solidification, often termed gel fixation, is essential for preserving filament geometry, retaining flow‐induced mesogen alignment, and ensuring structural stability immediately after deposition. Because LCEs' precursor inks exit the nozzle in a viscoelastic state, they remain vulnerable to spreading, sagging, jet breakage, or relaxation of director orientation prior to network locking [1, 6]. Rapid gelation transforms the filament from a deformable fluid into a mechanically coherent intermediate, preventing relaxation‐driven loss of anisotropy that would otherwise degrade actuation performance [37, 48]. In photoreactive LCEs chemistries based on acrylates, thiol‐ene reactions, and mesogenic diacrylates, ultraviolet exposure directly at the nozzle exit or just downstream is widely used to increase network modulus and immobilize orientation patterns generated during flow [7, 42]. UV‐induced polymerization stabilizes high‐aspect‐ratio filaments, reduces die swell, and suppresses gravitational deformation, enabling the preservation of uniform diameter throughout the print path [80, 81]. Nozzle‐mounted LED collars and ring‐shaped irradiation arrays allow dynamic matching of curing intensity to extrusion rate, permitting continuous deposition of free‐standing structures, helical trajectories, and programmed 4D‐printed geometries [42, 48]. UV‐assisted fiber drawing has also been successfully applied in LCEs filament manufacturing, where flow‐aligned microfibers maintain orientation across tens of centimeters, producing reproducible reversible strain along the fiber axis [42, 65, 81].

## 5 Orientation Fixation and Network Locking

**FIGURE 7:**
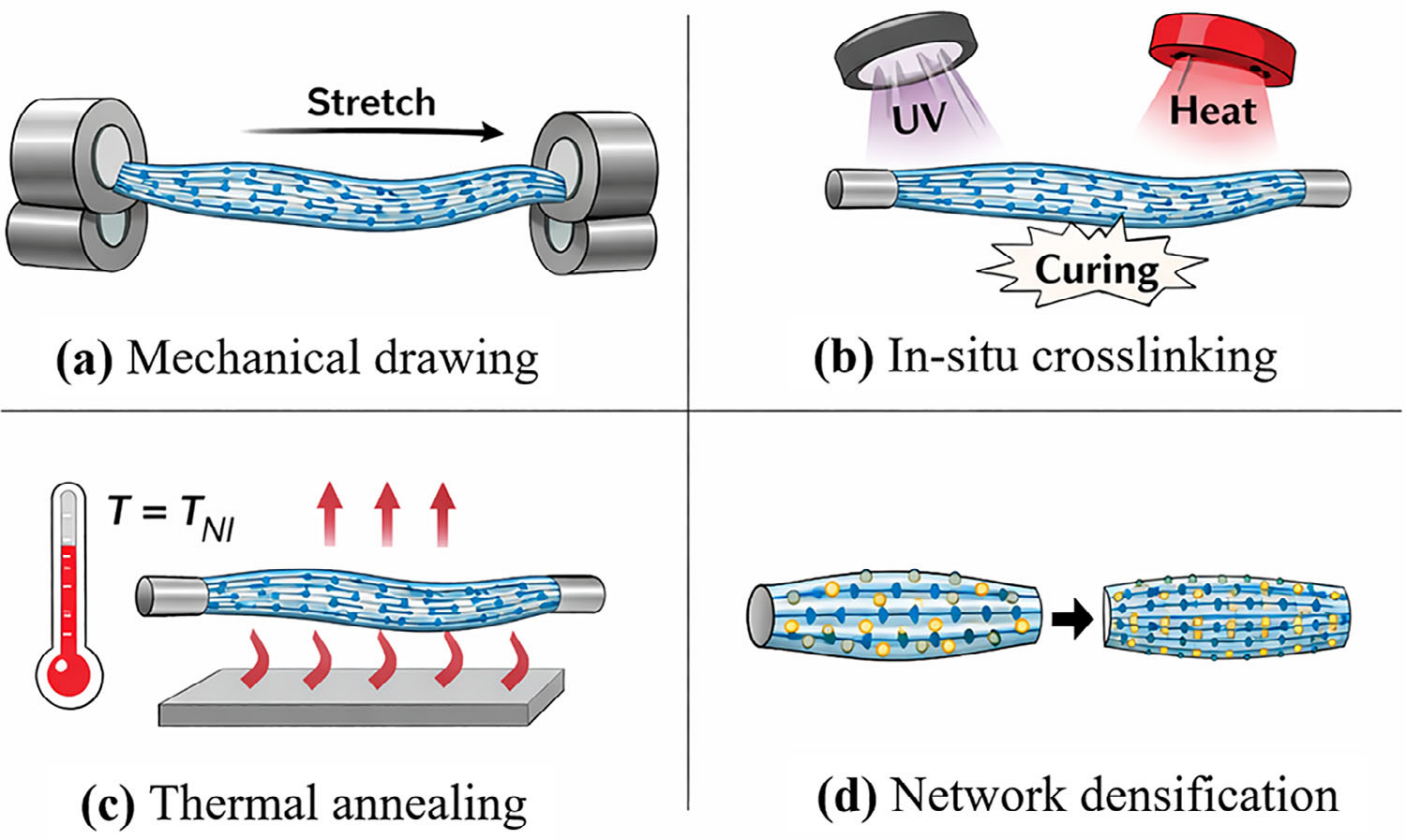
Orientation fixation strategies in liquid crystal elastomer filaments. Schematic overview of major approaches converting transient director alignment into permanent anisotropy: (a) mechanical drawing to amplify and homogenize director alignment; (b) in situ crosslinking by UV or thermal curing to immobilize orientation; (c) thermal annealing near the nematic‐isotropic transition to heal defects; and (d) network densification through secondary curing or solvent removal to stabilize long‐term performance.

After extrusion, spinning, or jetting, flow‐induced mesogen alignment remains metastable until the polymer network is locked through crosslinking, densification, or thermal reorganization. Orientation fixation is therefore the decisive step that determines the magnitude and durability of actuation strain, optical anisotropy, and mechanical performance of LCE architectures [1, 6]. Across DIW printing, wet‐spinning, melt‐drawing, and fiber fabrication, four primary strategies‐mechanical drawing (Figure 7a), in situ crosslinking (Figure 7b), thermal annealing (Figure 7c), and network densification (Figure 7d) are used to convert transient flow‐induced director fields into stable monodomain states [37, 48]. When properly coordinated, these mechanisms enable LCE filaments and woven or braided assemblies to sustain reversible, directionally specific shape change with minimal fatigue over long operating lifetimes [7, 42]. Recent comprehensive reviews, such as that by Zang and co‐workers [137], have provided critical insights into the emerging fabrication strategies for liquid crystal elastomers, emphasizing the transition from film‐based synthesis to scalable manufacturing platforms. These developments collectively define the current state‐of‐the‐art in LCEs research and underscore the importance of integrating chemistry, processing, and device‐level applications.

### 5.1 Mechanical Drawing

Mechanical drawing (post‐extrusion extension) is the most effective method for amplifying and homogenizing director alignment in LCE fibers. Tensile deformation imposes an extensional field along the filament axis, reinforcing alignment generated during nozzle shear and suppressing radial director gradients [1, 48]. Experimental studies consistently show that higher draw ratios increase actuation strain, tensile modulus, and elastic recovery in wet‐spun, melt‐spun, and antigelation‐spun fibers [20, 41]. Mechanical drawing also enables post‐process texture refinement in textile‐integrated actuators. When applied to woven or braided structures, tensile alignment promotes monodomain filament behavior, improving bending uniformity, torsional response, and fatigue resistance under load [57, 130]. Drawing is frequently combined with early‐stage partial crosslinking to prevent plastic deformation and to stabilize alignment as strain is applied [6, 37].

### 5.2 In Situ Crosslinking

In situ crosslinking immobilizes director alignment during or immediately after extrusion, drawing, or jet formation. Photocurable LCEs formulations‐acrylates, thiol‐ene networks, and reactive mesogenic diacrylates are commonly locked using UV irradiation at the nozzle exit or along a draw path, freezing shear‐ or extension‐induced anisotropy before relaxation can occur [7, 42]. UV‐assisted fiber drawing allows continuous fabrication of long, uniformly aligned filaments with large reversible thermal actuation [41]. Thermally or catalyst‐triggered curing plays a parallel role in hydrosilylation‐based elastomers, particularly during melt extrusion where elevated temperatures destabilize nematic order unless curing is synchronized with stretching [6, 37]. In coaxial spinning, selective in situ crosslinking of either the core or shell stabilizes multilayer architectures under high draw ratios and prevents interfacial slippage or collapse [58, 130].

### 5.3 Thermal Annealing

Thermal annealing is a post‐processing step that improves director uniformity and stabilizes functional anisotropy. Annealing near the nematic‐isotropic transition temperature increases mesogen mobility, enabling disclination relaxation and defect healing while maintaining the imposed director orientation [1, 48]. When performed after curing, annealing homogenizes the director profile across the fiber cross‐section, increases actuation strain, and reduces residual stress buildup, improving long‐term response reproducibility [6]. In textile‐integrated LCES systems, annealing compensates for damage from mechanical handling, weaving, or embroidery, restoring monodomain alignment and preventing spatially inconsistent strain during actuation [57, 130]. Similar improvements are observed in printed lattices and 4D‐architectures, where annealing eliminates local orientation discontinuities introduced during toolpath transitions [7, 42].

### 5.4 Network Densification

Network densification stabilizes director orientation by increasing crosslink density, suppressing local mesogen rotation, and reducing free volume. Densification can occur through secondary crosslinking, thermal post‐curing, solvent extraction, or crystallization‐driven packing [6, 37]. These mechanisms strengthen the polymer scaffold, reduce creep, and enhance fatigue resistance under cyclic strain. In wet‐spun filaments, solvent out‐diffusion during and after coagulation acts as an intrinsic densification stage, shrinking the filament and tightening the network; this effect increases mechanical robustness and improves high‐strain actuation [20, 45]. In melt‐drawn fibers, densification may coincide with LC‐domain crystallization or phase ordering, forming hybrid elastomer‐crystalline networks that produce high blocking forces and superior cycling stability [20, 41].

## 6 Applications of LCEs Filaments

The transition of LCEs from bulk films to continuous filamentary forms significantly broadens their application landscape by enabling direct integration into fiber‐based and textile architectures. This dimensional shift is particularly important for translating the intrinsic actuation capabilities of LCEs into scalable, device‐relevant formats. In soft robotics, LCE filaments function as lightweight, compliant actuators capable of reversible contraction, bending, and twisting in response to thermal, optical, or electrical stimuli [10, 11]. Their high strain capability and anisotropic response allow them to mimic biological muscles, making them suitable for applications such as soft grippers, locomotion systems, and adaptive robotic components. Compared to bulk LCE films, filament‐based architectures offer improved flexibility, faster response times, and easier integration into complex geometries.

In the field of smart textiles, LCE filaments can be woven, knitted, or embroidered into fabrics to create adaptive and responsive materials [13, 14]. These systems enable thermoresponsive ventilation, dynamic shape morphing, and haptic feedback in wearable devices. The compatibility of LCEs filaments with conventional textile processing techniques represents a major step toward scalable manufacturing of responsive garments and functional fabrics. Aligned LCE fibers are also highly promising for artificial muscle systems, where their combination of large reversible strain, low density, and programmable anisotropy enables efficient actuation [11, 20]. Such fibers can be assembled into bundles or hierarchical structures to amplify force output and achieve biomimetic motion, making them attractive for assistive devices, prosthetics, and bio‐inspired engineering systems.

Beyond actuation, LCES filaments enable multifunctional platforms when combined with conductive or photothermal fillers such as carbon nanotubes, graphene derivatives, or metallic nanoparticles [55, 56, 57, 58, 63, 64, 65]. These hybrid systems support electrothermal or light‐driven actuation while simultaneously enabling sensing, signal transduction, or energy conversion. For instance, CNT‐integrated LCE fibers exhibit efficient Joule heating and reversible contraction, whereas photothermal fillers allow remote, light‐triggered actuation with spatial control. Emerging applications also include optical waveguides, adaptive optics, and soft sensors, where the coupling between mechanical deformation and optical or electrical properties can be exploited [58, 59, 60]. In these systems, filament geometry provides advantages in guiding light, enhancing surface area, and enabling structural programmability. Overall, the development of LCE filaments bridges the gap between material design and real‐world implementation by enabling scalable, flexible, and multifunctional actuator systems. These applications underscore the importance of controlled processing strategies, as discussed in this review, to achieve reproducible alignment, mechanical integrity, and long‐term functional performance.

## 7 Finishing and Performance Stabilization

Following filament formation and orientation fixation, finishing treatments are required to ensure long‐term stability, reproducible actuation, and durable mechanical performance in liquid crystal elastomer fibers and printed structures. These treatments act on the microstructure that continues to evolve after initial network locking, and they are particularly important for devices exposed to repeated thermal or mechanical cycling. The most widely implemented strategies include post‐cure stabilization, mechanical conditioning, and surface or interface modification, each of which targets distinct degradation pathways and defect mechanisms in LCE architectures [6, 37].

Post‐cure stabilization refers to additional crosslinking or thermal treatment that completes polymer network formation and eliminates residual reactive groups. Secondary curing increases the elastic modulus, reduces creep, and improves dimensional stability under cyclic stress by tightening the network architecture and lowering defect mobility [6, 37]. In wet‐spun LCE fibers, solvent evaporation combined with programmed thermal post‐curing densifies the polymer network and sharpens monodomain alignment established during spinning or draw‐down. This leads to enhanced actuation strain and improved blocking force during prolonged cycling in textile use‐cases [20, 130]. In direct‐ink writing, post‐curing eliminates interlayer softness and increases cohesion across deposition interfaces, which enhances mechanical uniformity in 4D‐printed lattices and actuator frameworks [7, 80]. Mechanical conditioning further stabilizes the network by reorganizing mesogenic domains under controlled repeated actuation. Cyclic loading removes residual stresses formed during fabrication, lowers hysteresis, and redistributes microdefects across the network [7, 42]. Repeated photothermal or Joule heating cycles in LCE filaments substantially improve strain reproducibility and fatigue resistance by relaxing frozen stresses at LC domain boundaries [6, 48]. Textile‐integrated systems, including OmniFiber actuator yarns and embroidered LCEs assemblies, show higher symmetry in bending and steady‐state cycling performance after moderate preconditioning, highlighting the essential role of this step in practical deployments [57, 130].

Surface and interface modification is particularly important when LCE filaments are integrated into textiles, composites, or multi‐material architectures. Plasma activation, silane coupling, and polymer coating treatments improve interfacial adhesion and reduce slip at yarn junctions. These treatments protect fibers from abrasion, redirection of director fields, and stress concentration during repeated bending or embroidery processes [57, 130]. Surface coatings also mitigate environmental degradation, controlling moisture uptake and oxidation that shift actuation onset temperature and depress strain magnitude over time [6, 37]. Collectively, post‐curing, conditioning, and surface modification form the final essential stage of processing. They ensure that LCE filaments, printed structures, and textile actuators sustain stable anisotropy, consistent actuation, and mechanical reliability throughout their operational lifetime [7, 42].

## 8 Sustainability, Environmental Responsibility, and Standardization Needs

As liquid crystal elastomer technology moves toward pilot‐scale manufacturing, sustainability and regulatory compliance have become foundational design criteria. Conventional LCEs synthesis relies heavily on acrylate mesogens, tin or platinum catalysts, and volatile organic solvents, including dichloromethane and dimethylformamide. Each of these materials presents documented challenges with toxicity, worker exposure, solvent disposal, and environmental persistence [6, 37]. A transition toward greener chemistries is therefore gaining momentum, including solvent‐free formulations, low‐VOC resin systems, and photoinitiators designed for reduced ecotoxicity. Bio‐derived LC monomers from renewable aromatic feedstocks have been proposed to replace oil‐derived mesogens without compromising nematic phase stability [17, 53].

Dynamic covalent networks provide another path toward sustainability. Vitrimeric or exchangeable bond systems retain liquid‐crystalline order while allowing healing, reshaping, and depolymerization. This enables circular‐material models through reprocessing and feedstock recovery [53]. Such networks have particular relevance to LCEs fibers integrated into consumer textiles because they enable repair and recycling while maintaining programmable actuation behavior. Industrial responsibility must also address manufacturing infrastructure. Closed‐loop solvent recovery, catalyst filtration, and off‐gas purification systems are necessary to ensure safe production conditions and environmental compatibility [37]. As adoption expands, reproducibility becomes equally important. Currently, no standardized framework comparable to ASTM or ISO exists for LCE rheology, mesogen alignment, strain fatigue, or actuation performance metrics. Interlaboratory round‐robin testing, standardized metadata reporting including monomer ratios and curing dosage, and calibration of print parameters will be essential to achieving reproducible and certifiable products [6, 37]. These standards will be critical as LCE filaments enter regulated markets such as biomedical wearables, aerospace morphing systems, and haptic textiles.

## 9 Barriers, Open Problems, and Future Outlook

Despite rapid progress, several scientific and technological constraints still impede industrial adoption of LCE filaments. One central challenge is the absence of predictive models capable of capturing the coupled dynamics of nematic rheology, shear‐induced alignment, and photo‐curing kinetics. Current constitutive models describe director dynamics or viscoelasticity in isolation, but fully coupled multiphysics simulations integrating nematodynamics, curing, heat transfer, and structural relaxation remain under development [1, 48]. To address these Multiphysics modeling barriers, emerging data‐driven approaches, including ML and AI‐driven formulation optimization, offer a promising alternative to traditional constitutive modeling. By leveraging large experimental datasets of rheological profiles, curing kinetics, and actuation strains, ML algorithms can identify non‐linear correlations between molecular architecture and processing windows that are often too complex for purely analytical frameworks [138]. These AI‐assisted tools could potentially accelerate the discovery of optimal resin compositions and extrusion parameters, enabling real‐time feedback loops that compensate for material drift and ensure consistent director alignment during high‐throughput filament fabrication. These models are particularly important for continuous manufacturing processes such as fiber drawing and DIW where alignment, phase state, and curing occur simultaneously.

Material ageing presents another barrier. Humidity, oxygen ingress, and repeated thermal cycling degrade mesogen alignment, alter the nematic‐isotropic transition temperature, and reduce strain output. Real‐time metrology capable of detecting drift and feedback systems that compensate during manufacturing will be required to maintain reliability under extended operation [7, 42]. Process hardware must evolve concurrently. Industrial‐grade LCEs manufacturing demands stable extrusion pressure, uniform nozzle temperature, and micron‐scale calibration over sustained print times. Modular manufacturing cells integrating heated extruders, inert‐gas curing tunnels, annealing chambers, and optical inspection modules represent a promising path toward scalable systems [58, 130]. Emerging research highlights hybrid architectures that unify filament formation with structural functionality. Reinforced LCE composites incorporating continuous fibers improve mechanical durability under bending and torsion [120]. Wet‐spun LCE yarns integrate fluidic channels and optical sensing capabilities into textile actuators [57]. Multidomain actuators combining elastomer and crystalline LCEs phases enable high blocking forces and ultrafast responses suitable for robotics and wearable haptics [45, 57]. Recent perspectives [17] and the thirty‐year retrospective on LCEs science, emphasize that fundamental principles of actuation are established and that scalable manufacturing and standardization now constitute the primary bottleneck.

## 10 Conclusion

Scalable LCEs filament manufacturing requires an integrated framework that combines molecular formulation, rheological conditioning, filament formation, in situ curing, orientation fixation, and performance stabilization. Crucially, the coupled interplay between viscosity, extrusion pressure, and flow rate must be carefully controlled to ensure defect‐free filament formation and consistent alignment retention during scale‐up. RM82‐based systems with multi‐functional crosslinkers such as TATATO and two‐stage curing architectures currently provide robust processing windows, reliable alignment retention, and reproducible actuation suitable for textile and soft robotic applications. A comparative understanding of viscosity regimes further indicates that optimal filament quality and actuation performance are achieved within narrow intermediate processing windows, highlighting the importance of precise rheological tuning. LCEs' progress depend on standardized processing metrics and reporting protocols, including rheological characterization, extrusion parameters, curing kinetics, and cyclic durability testing, to improve reproducibility and enable industrial translation. In parallel, emerging data‐driven approaches, particularly machine learning‐assisted formulation and process optimization, offer significant potential to accelerate the discovery of composition processing performance relationships in LCE systems. Sustainable development will require recyclable dynamic networks and renewable mesogenic feedstocks. LCEs filaments uniquely connect molecular‐scale order with macroscopic mechanical function, positioning them as enabling materials for adaptive textiles, morphing robotic systems, and responsive interfaces. The convergence of advanced materials design, coupled process control, standardized metrology, and digital optimization tools will be essential for transitioning LCE filaments from laboratory‐scale demonstrations to scalable, reliable, and industry‐ready smart materials.

## Funding

This work was supported by Hochschule Niederrhein, University of Applied Sciences, Mönchengladbach.

## Conflicts of Interest

The authors declare no conflicts of interest.

## Data Availability

The data that supports the findings of this study are available in the supplementary material of this article.
